# Transgender Healthcare: Development of an Illustrated eLearning Tool for Medical Education

**DOI:** 10.15694/mep.2021.000159.1

**Published:** 2021-06-07

**Authors:** James Young, Jill Gregory, Mary Rojas, Gale Justin, Tamara Kalir

**Affiliations:** 1 Department of Medical Education; 2 Departments of Medical Education and Obstetrics

**Keywords:** Instructional Technology, Transgender Healthcare, eLearning, Teaching module, medical school curriculum

## Abstract

This article was migrated. The article was marked as recommended.

Purpose

Transgender health competency among medical students and clinical providers remains poor, yet standardized curricula are lacking. Integrating the rapidly evolving teaching methods of the current technological era, a team of physicians and instructional designers created and evaluated a visual-format, interactive eLearning module to teach core competencies of transgender healthcare.

Methods

From September-March 2020, 416 students (MS1-MS4) from a NY-based medical school participated in the curriculum, which covered sexual development, gender affirmation surgeries, medical management, and health screening for transgender patients. Students completed pre/post surveys about their knowledge, comfort, and preparedness. Changes were assessed using the Chi-squared test. Commentaries were evaluated with thematic analysis.

Results

Pre-intervention, 68% of MS4s and 53% of MS3s rated the preclinical transgender curricula as “very poor,” “poor,” or “fair.” Among the 187 students who took the module and post-survey, 79% felt “more comfortable” and 81% felt “more prepared” in providing healthcare to transgender patients after completion. Each class demonstrated statistically significant increases in comfort compared to baseline assessments. Students submitted >150 positive comments on the module’s educational content, illustrations, and functionality.

Conclusions

As medical schools increasingly embrace virtual learning, this interactive learning tool serves as a model for expanding transgender healthcare curricula throughout the country.

## Introduction

There is growing recognition of the need to expand transgender health curricula in medical schools throughout the United States. However, there is yet to be agreement on the exact interventions that should be used to address transgender health education ( Marshall, Pickle and Lawlis, 2017). Today, transgender curricula remain sparse and predominantly composed of social awareness-based lessons that lack topic-specific competencies ( Dubin *et al.*, 2018). Consensus in the existing literature supports educational approaches for transgender health teaching to shift toward learning initiatives that are longitudinally integrated, interactive, and clinical skills based ( Liang *et al.*, 2017).

A 2018 meta-analysis reveals that few studies have directly assessed medical student attitudes and knowledge about transgender patients and transgender health issues ( Dubin *et al.*, 2018). Of the few found, one 2016 study showed that 74% of medical students report receiving <2 hours of curricular time devoted to transgender clinical competency in all 4 years combined ( Dowshen *et al.*, 2016). A study at Boston University demonstrated that students reported lower knowledge and comfort with transgender and intersex health than lesbian, gay, and bisexual (LGB) health ( Liang *et al.*, 2017). An anecdotal review of the preclinical curriculum at the Icahn School of Medicine at Mount Sinai (ISMMS) found that the word “transgender” was included in only 8 of the 658 medical science lecture PowerPoints presented in 2016-2017, excluding the Art and Science of Medicine humanities course, performed using the word search function. This may be explained by schools’ disproportionate focus on LGB people, frequently discussed in the context of a “high-risk” or “specialty” population.

Transgender health education remains primarily restricted to smaller social science sessions, while cisgender identity is the assumed default in most medical science courses. Some medical schools are attempting to remedy this disparity by creating panels of mixed lesbian, gay, bisexual, transgender, queer, and intersex (LGBTQI+) patients and lessons on establishing a comfortable clinical environment for gender minorities. Although important, creating a respectful culture is insufficient without knowledge of the specific medical and surgical services available for transgender people ( Hollenbach, Eckstrand and Dreger, 2014). While previous studies demonstrate a clear need for increased curricular coverage of transgender healthcare, they expose two deficiencies in current research: 1. transgender and LGBQI+ individuals are frequently grouped together, when experiences, health needs, and educational objectives vary widely; and 2. few models exist to improve transgender health curricula ( Obedin-Maliver *et al.*, 2011). In order to create such a model, instructional technology may be employed to provide a multisensory learning experience.

One of the most powerful and longstanding educational tools in medicine is visualization. Images transcend language and communicate concepts quickly - vital to the demands of medical education. The direct sensory impact of images can improve attention, cognition, reflection, pattern recognition, and memory retention in ways that other learning modalities do not ( Norris, 2012). Medical schools are increasingly recognizing how multimedia-aided instruction can significantly shorten time needed to learn complex skills. More and more courses are being replaced by self-instructional courseware, which allows students to learn in their own time and location ( Ansary and El Nahas, 2000). This may explain the significant decline in classroom attendance across US medical schools, as more students opt for learning lecture content at home ( Murphy, 2019). Instructors also benefit from the ability to quickly and cost-effectively distribute virtual courses to a broader audience.Never has visual, computer-based learning been more relevant than today during the COVID-19 pandemic.

**Visual** teaching is especially well-suited to transgender healthcare, as many of the treatment options alter the physical body. Whether through hormone replacement therapy (HRT) or surgery, physical changes occur to the chest, pelvis, face, hair, and muscles. This alteration in anatomy is important to understand for healthcare providers when assessing routine health screening. There are few accurate and comprehensive visuals available to clinicians that illustrate the anatomical changes of transgender patients post-HRT or gender affirmation surgery. This translates to providers not knowing what questions to ask or exams to perform ( Dutchen, 2018). One study found that 50% of transgender people report having to teach their providers about transgender care, and 19% have been refused care due to their identity ( Grant *et al.*, 2019). With this awareness, we developed and tested the efficacy of a visual, interactive eLearning module to teach transgender healthcare to medical students at ISMMS.

## Methods

### Survey design and administration

This study was a pretest-posttest design of a curricular model assessing comfort and preparedness with transgender healthcare. Invited participants were medical students actively enrolled in years 1-4 at ISMMS during academic year 2019-2020. We obtained approval for this study from the ISMMS Institutional Review Board.

We asked the medical students to complete two anonymous surveys: one at the beginning of the academic year, pre-intervention, and one at the end of the academic year, post-intervention (see Supplementary File 1). Our surveys were modeled after questionnaires utilized by prior studies( Obedin-Maliver *et al.*, 2011; White *et al.*, 2015) and validated by the Center for Transgender Medicine and Surgery (CTMS) at Mount Sinai. The pre-survey assessed the following baseline data: 1) knowledge and comfort with 9 transgender-related healthcare domains; 2) perceived integration of pertinent transgender health education in courses taken thus far; 3) overall quality of transgender education at ISMMS; 4) preferred learning styles; and 5) demographics. The 9 healthcare domains assessed were: primary care for transgender patients, including health screening guidelines; medical interventions available for transgender patients, including HRT; gender affirmation surgeries, including pelvic and chest procedures; intersex healthcare and the relationship to transgender health services; barriers to medical care; social services available for transgender people; transgender adolescent health; mental healthcare; and reproductive healthcare for transgender patients, including fertility-preservation options.

The post-module survey included 3 components: 1) 20-point quiz on core transgender healthcare topics covered in the module, 2) assessment of module efficacy and changes to comfort and preparedness in providing medical care to transgender patients, and 3) qualitative free-text response to the module, without word limit. This survey measured changes in comfort with respect to 5 of the 9 transgender healthcare domains assessed in the pre-survey. The quiz included 7 questions, 1-2 from each module chapter, in mixed formatting: matching, multiple choice, and “check all that apply.” The questions evaluated core principles described by the World Professional Association for Transgender Health (WPATH)"Standards of Care" ( Coleman *et al.*, 2017).

Knowledge and attitude questions used a 5-point Likert scale (i.e. very good - very poor, fully comfortable - uncomfortable, full coverage - coverage not needed) with options to indicate “don’t know” or “decline to answer” for every question. Baseline surveys were distributed by paper in class to MS1s and MS2s and online to MS3s and MS4s. All post-module surveys were administered online.

### Module design and administration

To deliver the transgender education content, we created an interactive, illustration-based learning module entitled “Common Origins: Sex as a Spectrum and Transgender Healthcare.” This module was developed in collaboration with a diverse team: physician and course director of Sexual and Reproductive Health (TK), learning specialists (GJ, JG, MR), and instructional designer and medical illustrators (JG, JY). The module was created using Adobe Captivate, which is an authoring tool used to generate eLearning content like software demonstrations, simulations, and randomized quizzes in Small Web Formats (.swf) and HTML5 formats. The Captivate module can be integrated into learning management systems, like Blackboard, for student access.

The learning content was synthesized from multiple sources. Learning objectives were guided by an existing transgender health curriculum at ISMMS organized by TK. This curriculum had previously undergone a rigorous review process by the Department of Medical Education and Student Affairs at ISMMS. In this curriculum, students have access to a 20-page document entitled “Clinical Care of the Transgender Patient,” adapted from the NetCE Course #91920 ( Mesics, 2018). A second source of expertly-vetted content, the WPATH “Standards of Care for the Health of Transsexual, Transgender, and Gender-Nonconforming People, Version 7” was closely referenced for module development. Lastly, the module incorporated information provided by leading surgeons in the field of transgender health at Mount Sinai: Marci Bowers, MD, a pelvic and gynecologic surgeon recognized as the first transgender woman to perform transgender surgery ( Bowers, 2016); Rajveer Purohit, MD, a reconstructive urologist; and Jess Ting, MD, a plastic surgeon who specializes in gender affirmation surgery. All learning content followed the AAMC professional competencies for medical school education of sexual and gender minorities **( Hollenbach, Eckstrand and Dreger, 2014)**. A focus group of 4 medical and graduate students, 3 of whom identify as trans, reviewed and revised the final module before release to the school.

We deployed the module in the MS2 course “Sexual and Reproductive Health (SRH)” and opened it to MS3s and MS4s as an extra-curricular activity. MS1s were excluded, as they will take SRH the following year. The module has 4 learning objectives: 1) Expand understanding of sex, gender, and embryologic development; 2) Recognize how past management of differences of sexual development (DSD; Intersex) paved the way for medical and surgical procedures for transgender patients; 3) Recognize the importance of gender affirmation surgeries for transgender patients: penile-inversion vaginoplasty, metoidioplasty, phalloplasty, and chest reconstruction; and 4) Improve overall healthcare competency for transgender patients. Gender affirmation surgeries are emphasized to advance the learning of pelvic and breast anatomy and health screening guidelines.

The module contains 6 chapters: Embryology, DSD, Transwomen - Pelvis, Transmen - Pelvis, Chest, and Quiz. Pages are advanced by clicking on the forward or back arrows, or by clicking on the desired chapter button. A “Home” button takes the learner to Learning Objectives and Framework. An “Information” button takes the learner to References, Table of Contents, and Terminology. Images have roll-over functionality to reveal different anatomical views, supplementary labels or descriptions, and/or magnifications of important details. Embedded in the module are matching games and self-assessment checkpoints to establish learning retention. The module takes two hours to complete.

### Data Analysis

We compared average Likert responses across class years and between the pre-module and post-module surveys. For each class cohort, the percentage of baseline comfort with the transgender healthcare domains was calculated by adding the total number of “comfortable” and “somewhat comfortable” responses and dividing by total number of responses, including “uncomfortable,” “somewhat uncomfortable,” “neutral,” “comfortable,” “somewhat comfortable,” “don’t know,” and “decline to answer.” The percentage of post-module positive change in comfort was calculated the same way, with the number of “more comfortable” and “somewhat more comfortable” responses divided by total number of responses. We performed statistical analysis of post-intervention positive change in comfort for each cohort using the “N-1” Chi-squared test as recommended by Campbelland Richardson (U.K.). P-values were calculated. For the post-survey quiz, we calculated the median score and semi-interquartile range. We analyzed free-text commentary by tallying and dividing responses into the 3 major themes discussed. Responses with comments on >1 theme were included in both categories. Responses were assessed for both positive and negative feedbackand categorized accordingly.

## Results/Analysis

### Demographics and pre-survey results

There were 578 total medical students enrolled in ISMMS at time of the study: 140 MS1, 140 MS2, 150 MS3, 148 MS4. Of these, 416 medical students enrolled in the study and completed the pre-survey in Fall 2019 in the following percentages: 69% MS1, 82% MS2, 51% MS3, and 86% MS4. The demographics of respondents are listed in Table 1.

**Table 1.** Characteristics of medical students who enrolled in the study and submitted the baseline survey

Characteristic	MS1: # (%) n = 97	MS2: # (%) n = 115	MS3: # (%) n = 76	MS4: # (%) n = 128	Total: # (%) n = 416
Age (years)					
18-25	86 (88.7%)	94 (81.7%)	43 (56.6%)	25 (19.5%)	248 (59.6%)
26-30	8 (8.2%)	17 (14.8%)	31 (40.8%)	93 (72.7%)	149 (35.8%)
31+	3 (3.1%)	2 (1.7%)	1 (1.3%)	5 (3.9%)	11 (2.6%)
Decline to answer	0 (0.0%)	2 (1.7%)	1 (1.3%)	5 (3.9%)	8 (1.9%)
Race/Ethnicity (multiple categories could be selected)					
Black or African-American	13 (13.4%)	11 (9.6%)	5 (6.6%)	11 (8.6%)	40 (9.6%)
East Asian	10 (10.3%)	23 (20.0%)	6 (7.9%)	6 (4.7%)	45 (10.8%)
Hispanic or Latino	15 (15.5%)	6 (5.2%)	5 (6.6%)	11 (8.6%)	37 (8.9%)
South Asian	12 (12.4%)	17 (14.8%)	18 (23.7%)	12 (9.4%)	59 (14.2%)
White	55 (56.7%)	51 (44.3%)	39 (51.3%)	76 (59.4%)	221 (53.1%)
Other ^*^	2 (2.1%)	5 (4.3%)	1 (1.3%)	4 (3.1%)	12 (2.9%)
Decline to answer	0 (0.0%)	9 (7.8%)	5 (6.6%)	14 (10.9%)	28 (6.7%)
Gender Identity (multiple categories could be selected)					
Female, cisgender	64 (66.0%)	54 (47.0%)	44 (57.9%)	61 (47.7%)	223 (53.6%)
Female, transgender	0 (0.0%)	0 (0.0%)	0 (0.0%)	0 (0.0%)	0 (0.0%)
Gender non-conforming	1 (1.0%)	0 (0.0%)	1 (1.3%)	1 (0.8%)	3 (0.7%)
Male, cisgender	30 (30.9%)	53 (46.1%)	29 (38.2%)	63 (49.2%)	175 (42.1%)
Male, transgender	2 (2.1%)	1 (0.9%)	0 (0.0%)	0 (0.0%)	3 (0.7%)
Non-binary	1 (1.0%)	1 (0.9%)	0 (0.0%)	2 (1.6%)	4 (1.0%)
Decline to answer	0 (0.0%)	6 (5.2%)	3 (3.9%)	2 (1.6%)	11 (2.6%)
Sexual Orientation (multiple categories could be selected)					
Asexual	1 (1.0%)	1 (0.9%)	0 (0.0%)	0 (0.0%)	2 (0.5%)
Bisexual	7 (7.2%)	5 (4.3%)	4 (5.3%)	6 (4.7%)	22 (5.3%)
Gay	6 (6.2%)	2 (1.7%)	5 (6.6%)	15 (11.7%)	28 (6.7%)
Lesbian	1 (1.0%)	0 (0.0%)	1 (1.3%)	3 (2.3%)	5 (1.2%)
Queer	4 (4.1%)	3 (2.6%)	3 (3.9%)	3 (2.3%)	13 (3.1%)
Questioning	3 (3.1%)	3 (2.6%)	2 (2.6%)	4 (3.1%)	12 (2.9%)
Straight	77 (79.4%)	95 (82.6%)	62 (81.6%)	96 (75.0%)	330 (79.3%)
Decline to answer	0 (0.0%)	8 (7.0%)	2 (2.6%)	5 (3.9%)	15 (3.6%)

* “Other” race/ethnicity responses included American Indian or Alaska Native (4), Middle Eastern (6), and Native Hawaiian or other Pacific Islander (1).

In the pre-survey, medical students expressed low levels of comfort and preparedness with transgender healthcare and a need to learn more. As an aggregate, MS1-MS4s felt *least* comfortable with gender affirmation surgeries and medical interventions for transgender patients. MS1-MS4s were *most* comfortable with understanding barriers to medical care. Pre-intervention, 68% of MS4s and 53% of MS3s rated the preclinical transgender health curricula to be either “very poor,” “poor,” or “fair.”

### Post-module survey results

**Figure 1.**
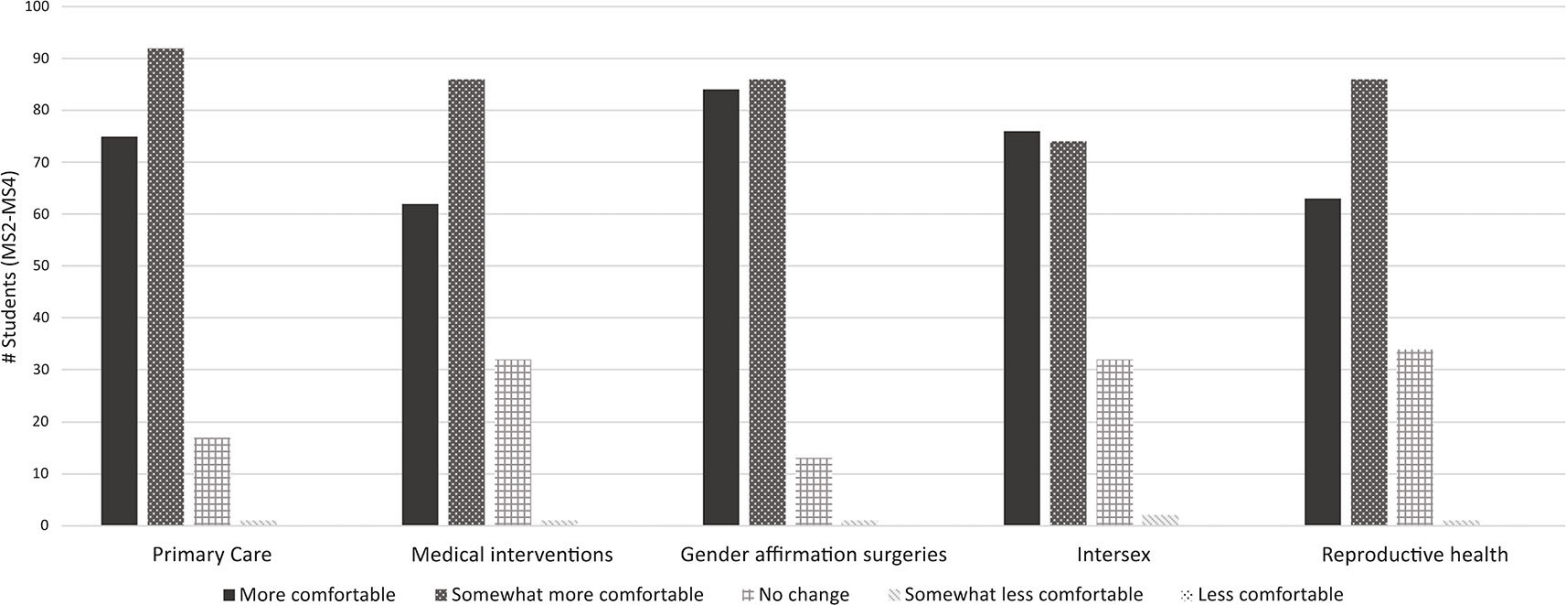
Change in MS2-MS4 Comfort with 5 Transgender Healthcare Domains, Evaluated After Module Completion

A total of 187 medical students in years 2-4 completed the module and post-module survey in March 2020, in the following percentages: 33% MS2, 28% MS3, and 40% MS4. The median score for the 20-point content-specific quiz was 17/20 (85%) with the semi-interquartile range of 15, 18. When rating the overall teaching efficacy on a 5-point scale, 92% of MS2-MS4s evaluated the module as “5/5: very helpful” or “4/5: moderately helpful.” As a result of the module, 79% of students reported feeling “more comfortable” in providing medical care to transgender patients, while 81% reported feeling “more prepared.” The majority of students reported feeling “more comfortable” or “somewhat more comfortable” with every transgender healthcare domain after completion of the learning module: Primary care, Medical interventions, Gender affirmation surgeries, Intersex healthcare, and Reproductive healthcare for transgender patients ( Figure 1).

* Change in student comfort was self-assessed using a 5-point Likert scale.

† Options for “don’t know” (n=6) and “decline to answer” (n=0) are not shown due to minimal responses.

Separated by class year, each medical student cohort demonstrated statistically significant increases in comfort with the 5 domains ( Table 2). For MS2, there was a 19% comfort rating pre-module, compared to 82% positive change in comfort post-module (Chi-square 305.7, P < 0.001). For MS3, there was a 40% comfort rating pre-module, compared to 84% positive change in comfort post-module (Chi-square 128.7, P < 0.001). For MS4, there was a 39% comfort rating pre-module, compared to 89% positive change in comfort post-module (Chi-square 226.8, P < 0.001).

**Table 2.** Pre- and Post-module Comfort with Transgender Healthcare

	Pre-survey		Post-survey			
	% comfort	n	% increased comfort	n	Chi-square	P-value
MS2	19	527	82	290	305.7	< 0.001
MS3	40	369	84	285	128.7	< 0.001
MS4	39	639	89	343	226.8	< 0.001

Number and percentage of medical students, separated by class year, who reported feeling comfortable with transgender healthcare on the pre-survey and increased comfort post-intervention. Chi-square and P-values assessing change in comfort for each cohort are shown.

### Student free responses to module

The 3 major themes discussed in student comments were module educational content, module illustrations, and module functionality ( Table 3). In total, there were 91 responses that directly addressed the module’s educational content, 53 that discussed the illustrations, and 23 that discussed the module’s functionality. For educational content, 89/91 of the responses spoke positively of the learning material, scope of curriculum, and specific language used to communicate the subject matter; 2 responses critiqued the curriculum, wanting more information on HRT and less on surgery. For illustrations, 52/53 responses spoke positively of the aesthetic quality, design, and sensibility of the images. One student wanted more illustrations on metoidioplasty and DSD. For module functionality, 22/23 responses expressed positive feedback to the user-friendly design with interactive inlays, embedded self-assessments, color-coding, and navigation tools. However, one MS2 wished there was an alternative format to JavaScript, which they commented is “not friendly for people with visual disabilities.” Sample comments are listed in Table 3.

**Table 3.** MS2-MS4 responses to the module entered in a free-text box at the end of the post-module survey

Major themes discussed	# responses on theme	Sample student responses:
Educational content or curriculum	91	“I appreciate that you go beyond terminology and actually get into the nitty-gritty of gender affirmation surgery; it’s not something most of us will ever get to see in an OR so it’s great to have it broken down. Understanding the operations on a basic level will allow us to take better care of patients who have undergone gender affirmation surgery. Starting with embryology provides an excellent framework for learning the operations included in this module. The level of detail is appropriate given that this is geared towards all medical students and not meant to be a surgical textbook.” - MS3
		“This module [is] thorough but concise, and all content that I feel like physicians should have familiarity with, regardless of specialty.” - MS4
Illustrations	53	“Fantastic illustrations with very clear labeling. This is the first module that goes into detail about transgender surgeries that I have seen, and it was incredibly helpful in picturing those procedures and enumerating the differences.” - MS4
		“The illustrations are easily some of the most clear/understandable anywhere. Beautifully done.” - MS2
		“The illustrations were so helpful. I wish I had those images when I was preparing for Step 1!” - MS3
Module functionality	23	“Very user-friendly, well-organized, and easy to navigate. I liked being able to roll over portions of images to get more information.” - MS2
		“The drawings with hover-over effects are amazing and clear. Facilitated my understanding a LOT. I love how color-coded everything is!” - MS2
		“The diagrams really make the material come to life.” - MS3

* Student comments were in response to the query: “If you have any thoughts or comments about this module, please share in the space below” (no word limit).

## Discussion

Despite increased initiatives to expand transgender healthcare across US hospitals, there remains pervasive discomfort and lack of knowledge with transgender medicine among medical students. Our pre-survey revealed low levels of student comfort and satisfaction with transgender healthcare teaching at ISMMS, which is consistent with the attitudes measured at other medical schools in prior studies ( Liang *et al.*, 2017; White *et al.*, 2015). These observations can be elucidated by examining the current teaching model.

When medical students take Anatomy, they frequently learn gender and sex as complementary binaries rather than independent spectra. They learn that XX represents “female” and XY represents “male,” with the implication that gender equates with biological sex. These associations can cause confusion when a patient’s gender does not match medically-expected sex chromosomes or genitalia. Students learn classic embryological development as two dichotomous paths, with less emphasis on common origins or the many variations that may arise. This construction of polarity can cause confusion when students encounter differences in sexual development or gender affirmation surgeries that rely on knowledge of XX-XY homologous pelvic structures.

The teaching module we developed aimed to transform existing curricula into an engaging platform that provides a comprehensive overview of sex as a spectrum and the medical and surgical services available today for transgender people. The majority of medical students evaluated the module as highly efficacious and felt more comfortable and prepared in providing medical care to transgender patients after completion. The students’ high performance on the quiz is consistent with their self-assessed positive changes in comfort and preparedness. Students cited a range of reasons to support the module’s efficacy: the ability to learn at one’s own pace, illustration-dominant learning format, interactive functionality, attention to language supplementing the images, and interconnections to other health disciplines. Even though transgender health content has previously been taught in coursework at ISMMS, the material has been presented in dense documents that are optional and minimally tested. This likely contributed to the student perceptions of inadequate transgender health teaching.

The module’s efficacy may be partly explained by the principles of Cognitive Theory of Multimedia Learning (CTML), which guided its development: 1. Multimedia principle: students learn better from words with images than from words alone; 2. Spatial contiguity principle: students learn better when corresponding words and images are presented near, rather than far from, each other; 3. Temporal contiguity principle: students learn better when corresponding words and images are presented simultaneously rather than successively; and 4. Coherence principle: students learn better when extraneous words, images, and sounds are excluded ( Mayer, 2009). In addition, the integration of diverse disciplines - from surgeons and medical doctors, instructional designers, and students of trans experience - was integral to building a comprehensive and user-friendly learning tool.

We acknowledge limitations to this study. One limitation was an inability to account for response bias. Student recognition of the need for increased transgender healthcare education may have influenced the significant positive responses to the post-module survey. Because the sample is NYC-based, there is likely a bias in favor of transgender inclusivity and expansion of transgender education. Even though the students at ISMMS come from diverse regions, the results may not be representative of other medical student populations across the country. It is important to note that while the pre-survey asked for baseline comfort with the transgender healthcare domains, the post-survey asked for *change* in comfort. Although the post-module increased comfort values are large, it is possible that students who said no change already felt comfortable with the material, deflating these values.

A second limitation is the lack of objective knowledge data from medical students prior to the module. While the post-survey integrated a transgender healthcare content quiz, the pre-survey strictly assessed subjective comforts and perceptions of educational curricula. While the minimal transgender health teaching in preclinical and clinical coursework suggest the quiz results are attributable to the learning module, there are insufficient data to show objective knowledge-based improvement as sole result of the module. A future follow-up study should be administered to assess information retention across academic years.

## Conclusion

While transgender-inclusive medical clinics expand throughout New York City and other metropolitan areas, education lags behind. This study reveals that an interactive, web-based visual learning module offers a promising solution to this discrepancy. Medical students at ISMMS responded with overwhelming support of the module and demonstrated improvements in knowledge, comfort, and preparedness after completion. This module serves as a model for expanding medical school curricula throughout the country to properly train student doctors to meet the needs of a population growing in visibility.

## Take Home Messages

• Transgender healthcare curricula in medical schools remain limited.
• Medical students and clinical providers report low levels of comfort, preparedness, and knowledge of transgender health services.
• The COVID-19 pandemic has underscored the need for well developed, illustrative virtual learning.
• We developed an interactive eLearning module on transgender healthcare that demonstrates robust evidence for improving student doctor training.

## Notes On Contributors

**James Young**, MD, is a graduate of the Icahn School of Medicine at Mount Sinai, NY, NY. He studied Science and Society at Brown University and Illustration at the Rhode Island School of Design. He is now a Urology resident at UCLA.

**Jill Gregory**, MFA, is research coordinator, certified medical illustrator, and Associate Director of Instructional Technology Group, Icahn School of Medicine at Mount Sinai, NY, NY. She has over 20 years’ experience as a medical and scientific illustrator.

**Mary Rojas**, PhD, is associate professor and Director of Medical Student Research Office, Icahn School of Medicine at Mount Sinai, NY, NY. She has over 30 years’ experience in quality improvement, education, and health services research, and formerly served as the Epidemiologist for Empire Blue Cross Blue Shield and Senior Director of Data Analysis at IPRO.

**Gale Justin**, PhD, is research supervisor and Director of Instructional Technology Group, Icahn School of Medicine at Mount Sinai, NY, NY. As a teaching expert, she has directed numerous education centers, formerly serving as Chief Learning Officer at Build Academy and Director of Instructional Technology at multiple academic institutions in NY.

**Tamara Kalir**, MD, PhD, is associate professor, research supervisor, Director of the Division of Gynecologic Pathology, and Course Director for Sexual and Reproductive Health, Icahn School of Medicine at Mount Sinai, NY, NY. She has over 35 years of clinical, educational, and research experience, with a focus on gynecologic pathology.

## References

[ref1] Ansary A. and El Nahas M. (2000) Medical Illustration in the UK: its current and potential role in medical education. Journal of Audiovisual Media in Medicine. 23(2), pp.69–72. 10.1080/01405110050010859 10912327

[ref2] Bowers M. (2016) Aesthetic male-to-female transsexual surgery. Female Genital Plastic and Cosmetic Surgery.pp.120–130. 10.1002/9781118848500.ch13

[ref3] Cohen R. D. (2019) Medical Students Push For More LGBT Health Training To Address Disparities, NPR. Available at: https://www.npr.org/sections/health-shots/2019/01/20/683216767/medical-students-push-for-more-lgbt-health-training-to-address-disparities( Accessed: 30 July 2019).

[ref4] Coleman E. Bockting W. Botzer M. Cohen-Kettenis P. (2017) Standards of Care for the Health of Transsexual, Transgender, and Gender-Nonconforming People. Principles of Gender-Specific Medicine.pp.69–75. 10.1016/B978-0-12-803506-1.00058-9

[ref5] Dowshen N. Meadows R. Byrnes M. Hawkins L. (2016) Policy Perspective: Ensuring Comprehensive Care and Support for Gender Nonconforming Children and Adolescents. Transgender Health. 1(1), pp.75–85. 10.1089/trgh.2016.0002 28861528 PMC5549535

[ref6] Dubin S. N. Nolan I. T. Streed C. G. Jr Greene R. E. (2018) Transgender health care: improving medical students’ and residents’ training and awareness. Advances in Medical Education and Practice.Volume9, pp.377–391. 10.2147/AMEP.S147183 29849472 PMC5967378

[ref7] Dutchen S. (2018) Transforming Transgender Care, Transforming Transgender Care, Harvard Medical School. Available at: https://hms.harvard.edu/news/transforming-transgender-care( Accessed: 3 September 2019)

[ref8] Encandela J. Zelin N. S. Solotke M. and Schwartz M. L. (2019) Principles and Practices for Developing an Integrated Medical School Curricular Sequence About Sexual and Gender Minority Health. Teaching and Learning in Medicine. 31(3), pp.319–334. 10.1080/10401334.2018.1559167 30661414

[ref9] Grant J. M. Mottet L. A. Tanis J. Harrison J. (2019) Injustice at Every Turn: A Report of the National Transgender Discrimination Survey (2019), National LGBTQ Task Force. Available at: https://www.thetaskforce.org/injustice-every-turn-report-national-transgender-discrimination-survey/( Accessed: 3 September 2019).

[ref10] Hollenbach A. D. Eckstrand K. L. and Dreger A. D. (2014) Implementing curricular and institutional climate changes to improve health care for individuals who are LGBT, gender nonconforming, or born with DSD.: A resource for medical educators. Association of American Medical Colleges. ( Accessed: 3 August 2019).

[ref11] Liang J. J. Gardner I. H. Walker J. A. and Safer J. D. (2017) Observed Deficiencies In Medical Student Knowledge Of Transgender And Intersex Health. Endocrine Practice. 23(8), pp.897–906. 10.4158/EP171758.OR 28534684

[ref12] Marshall A. Pickle S. and Lawlis S. (2017) Transgender Medicine Curriculum: Integration Into an Organ System-Based Preclinical Program. MedEdPORTAL. 13(1). 10.15766/mep_2374-8265.10536 PMC6342291 30800738

[ref13] Mayer R. E. (2009) Multimedia learning. Cambridge: Cambridge University Press.

[ref14] Mesics S. (2018) Clinical Care of the Transgender Patient - NetCE. Available at: https://www.netce.com/learning.php?page=coursedetails;courseid=1661( Accessed: 5 May 2019).

[ref15] Murphy B. (2019) Why some medical students are cutting class to get ahead, American Medical Association. Available at: >https://www.ama-assn.org/residents-students/medical-school-life/why-some-medical-students-are-cutting-class-get-ahead( Accessed: 3 September 2019).

[ref16] Norris E. M. (2012) The constructive use of images in medical teaching: a literature review. JRSM Short Reports. 3(5), pp.1–8. 10.1258/shorts.2012.011158 22666530 PMC3365786

[ref17] Obedin-Maliver J. Goldsmith E. S. Stewart L. White W. (2011) Lesbian, Gay, Bisexual, and Transgender-Related Content in Undergraduate Medical Education. JAMA. 306(9). 10.1001/jama.2011.1255 21900137

[ref18] Shergill A. K. Camacho A. Horowitz J. M. Jha P. (2019) Imaging of transgender patients: expected findings and complications of gender reassignment therapy. Abdominal Radiology. 44(8), pp.2886–2898. 10.1007/s00261-019-02061-9 31154481

[ref19] White W. Brenman S. Paradis E. Goldsmith E. S. (2015) Lesbian, Gay, Bisexual, and Transgender Patient Care: Medical Students. Preparedness and Comfort’, Teaching and Learning in Medicine. 27(3), pp.254–263. 10.1080/10401334.2015.1044656 26158327

[ref20] Zelin N. S. Hastings C. Beaulieu-Jones B. R. Scott C. (2018) Sexual and gender minority health in medical curricula in new England: a pilot study of medical student comfort, competence and perception of curricula. Medical Education Online. 23(1), p.1461513. 10.1080/10872981.2018.1461513 29717635 PMC5933287

