# Supplementary material for: Transgender Healthcare: Development of an Illustrated eLearning Tool for Medical Education
Source: MedEdPublish (2016). 2021 Jun 7;10:159. [Version 1] doi: 10.15694/mep.2021.000159.1 (PMC10939568; doi:10.15694/mep.2021.000159.1)
Supplement: Peer review history

# Reviewer report: Reviewer response for version 1

**DOI:** 10.21956/mep.18908.r27021

**Published:** 2021-10-12

**Authors:** Ken Masters

**Affiliations:** 1 Sultan Qaboos University

This review has been migrated. The reviewer awarded 3 stars out of 5 An interesting paper on the development of an illustrated eLearning Tool for transgender medical education.The background literature review establishes the clear need for increasing teaching about transgender and related medical issues in the USA. Similarly, the time is ripe for increased development of online learning tools.I am also pleased that the authors supplied a copy of their form and quiz, which have been well-constructed. (A small aside, related to the quiz in the supplementary file, not the paper: It is generally considered poor question design in an MCQ to ask students for the FALSE answer. This is especially problematic for students using a non-mother-tongue, because the FALSE answer is the CORRECT answer. So, in future quizzes, it would be advisable to re-write these questions).The study has been well-conducted, and will help the institution in the development of further material. As a research paper, though, the Discussion should have related the results back to the literature context outlined in the Introduction (this is done in only a single sentence at the beginning of the Discussion, but that is not nearly enough); otherwise, the relevance and importance of the study to readers outside the institution are not immediately apparent. (Perhaps, if the paper had been billed in the category of a New Educational Tool, that would have been more appropriate, and the expectations would have been different).Again, not related to the paper, but rather the module: The module itself could do with some work. Although it is online, and the graphic and text combination is good, it is a very simple click-through module reminiscent of early 1980s computer-based learning, that could simply be a standard paper-based textbook. I did see simple displays of labels on mouse-over and one drag-and drop exercise, but that was all. (If there was more, then it was not immediately obvious). So, for Version 2 of the module, I would recommend that the module be substantially re-vamped so that the real power of the media can be harnessed. (Oh, and please cut the sound (or at least give the user the option to turn it off) every time a button is clicked. It becomes really irritating and adds nothing to the learning process).So, I think the study has been well-done, but the authors should have substantially increased the value of their paper as a research paper by positioning it within the current literature.

Reviewer Expertise:

NA

No decision status is available

# Reviewer report: Reviewer response for version 1

**DOI:** 10.21956/mep.18908.r27020

**Published:** 2021-07-07

**Authors:** Anita Samuel

This review has been migrated. The reviewer awarded 4 stars out of 5 This is a very well-written article addressing a growing need in medical education. The Introduction provides a comprehensive overview of the current scenario regarding transgender healthcare and justifies the need for the study. The Methods section describes in detail the development of the interactive module and the survey instrument used. The strength of this article lies in the evidence-based foundation for this module and the explicit justifications provided for the choices made in this study. The reference to guiding theories in the Discussion is also appreciated. A few minor suggestions:- In the Abstract – Methods, you say September-March 2020. Stating September 2019 might be helpful.- In the Abstract and later in the article you refer to NY and NYC. Considering the international audience of this journal, providing the full form for these abbreviations is recommended.- A little more clarity on timelines would be helpful. For example, the module is 2 hours long. How long did the learners have access to this module?- It sounds like there might have been a gap between completing the module and the post-intervention survey. Some explanation about this would be helpful.

Reviewer Expertise:

NA

No decision status is available
